# Does early non-familial child care impact the development of mental health problems, risk behaviors, and educational outcomes until young adulthood?—Findings from an 18-year longitudinal study

**DOI:** 10.1186/s40359-026-04660-w

**Published:** 2026-05-05

**Authors:** Wolfgang Schulz, Ann-Katrin Job, Kurt Hahlweg, Max Supke

**Affiliations:** 1 Institute of Psychology, Technische Universität Braunschweig, Humboldtstr. 33, Brunswick, 38106 Germany; 2 Institute of Psychology, Universität Kassel, Kassel, Germany; 3 Leibniz Institute for Resilience Research (LIR), Mainz, Germany

**Keywords:** Early non-familial childcare, Mental health problems, Family, Young adulthood, Longitudinal study

## Abstract

**Background:**

International studies exploring the enduring effects of early non-familial childhood care on mental health outcomes show varying impacts depending on the quality of care, age of entry, and socio-economic factors.

**Methods:**

This German longitudinal study, involving 225 families, investigated the impact of early non-familial child care on the development of mental health problems, risk behaviors, and academic as well as vocational achievements in young adulthood (*M*_age_ = 22.6 years, *SD*_*age*_ = 1.1 years).

**Results:**

In comparison to parental care, early non-familial childcare was associated with significantly elevated levels of risky behaviors (such as risky sexual behavior, alcohol abuse, and tobacco consumption), low intimate relationship quality, and a higher likelihood of achieving advanced vocational qualifications. Migration background was identified as a relevant moderating factor in an exploratory analysis. An early age of entry into non-familial care was weakly associated with more mental health problems in young adulthood.

**Conclusion:**

Parental decisions on early non-familial childcare should carefully consider the child’s age of entry, the quality of care, and the family's socio-economic context.

**Supplementary Information:**

The online version contains supplementary material available at 10.1186/s40359-026-04660-w.

## Introduction

Non-familial early care of children up to school entry has increasingly become a focus of public attention and policy in recent decades. The care of children under three years old is particularly important. The establishment of more non-familial early childhood care (NFECC) facilities and the improvement of care quality, e.g., through an increase in caregiver-to-child ratios or the recruitment and permanent employment of qualified professionals, aim to contribute to the reduction of educational inequalities and to enhance the reconciliation of work and family life. In 2002, the European Council, e.g., set objectives that, by 2010, 33% of children under 3 years and 90% of children aged 3 to 6 years (school entry age) should be provided external non-familial care in European Union (EU) member states (“Barcelona Objectives”). This goal was already achieved for children under 3 years in 2017 (34.2%), although substantial variations exist among member states (e.g., < 10% in Bulgaria, Slovakia, Czech Republic, > 60% in Denmark, Luxembourg, Netherlands). For children aged 3 to 6 years, the rate in the EU was 93.3% in 2017 ([1]; see also [2]). Similar rates are observed in countries belonging to the Organization for Economic Cooperation and Development (OECD) [3]. In Germany, the proportion of children under 3 years in external non-familial care was 36% in 2023 [4], which is in line with the OECD and EU averages. Apart from these societal and political efforts, the advantages and disadvantages of NFECC are being discussed. It is debated that early and high-quality child care facilities are a crucial prerequisite for couples when making generative decisions or that, from an economic perspective, these facilities are increasingly important for the integration of young mothers and fathers into the labor market. However, what impact does this have on children? While non-familial care for children over 3 years of age is generally accepted and considered developmentally beneficial, there is still a controversial debate about non-familial care for those under 3 years of age.

In NFECC, a distinction is made between institutional care (such as daycare centers) and care provided by individual day care providers (daycare mothers or fathers). In the following, NFECC includes both types of non-familial child care.

### Child care research

In several longitudinal studies (e.g., (1) U.S. NICHD Study of Early Child Care and Youth Development, https://www.nichd.nih.gov/; (2) EPPSE Project in the United Kingdom, https://www.ucl.ac.uk/ioe/research-projects/2022/nov/effective-pre-school-primary-and-secondary-education-project-eppse2024; (3) MoBa Study in Norway, https://www.fhi.no/en/ch/studies/moba/) and meta-analyses (e.g. [5, 6]), predominantly from the United States, the psychological and social effects of NFECC on children, as well as the monetary costs and benefits, have been investigated.

In a more recent meta-analysis, Van Huizen and Plantenga examined 30 studies conducted between 2005 and 2017 in the USA, Canada, and Western Europe [7]. The meta-analysis exclusively included studies investigating universal NFECC programs. A broad spectrum of child outcome variables was assessed, encompassing cognitive and non-cognitive abilities, skills, academic performance, and labor market outcomes. The key findings can be summarized as follows: Overall, the results show considerable variability, with both positive (34.8%) and negative effects (21.7%), although the largest proportion of findings remained non-significant (43.5%). Neither the age at which children entered care nor the extent of care (part-time versus full-time) appeared to significantly impact the success of care. Instead, the quality of care emerged as the most important determining factor. Furthermore, long-term outcomes of NFECC – particularly in terms of educational attainment and labor market success – tend to be more favorable than the immediate effects observed in early childhood. Finally, children from disadvantaged backgrounds are more likely to benefit from NFECC, whereas for children from higher socioeconomic backgrounds, the likelihood of positive and negative effects appears to be roughly equal.

In their review, Zemp, Bodenmann and Zimmermann [8] came to a similar conclusion: The quality of care is crucial, including factors such as high continuity of care, a low caregiver-to-child ratio, manageable group sizes, and good education and qualifications of the childcare staff. The influence of the extent of care and age of entry, however, is controversially discussed, because there are positive and negative effects depending on the quality of care. Above all, the authors emphasize, that intrafamilial factors (e.g., parental sensitivity, a positive parent–child relationship, household income) are far more important for healthy child development than parameters of NFECC.

A recent German study based on data from the National Educational Panel Study (*N* = 1,559) found that higher levels of NFECC in children under three years of age were associated with lower rates of peer problems [9]. Additionally, for children with moderately difficult temperaments, higher rates of prosocial behavior were observed.

A Canadian longitudinal study, the Québec Longitudinal Study of Child Development, found significant effects of NFECC even at the age of 17 years [10]. Adolescents who had experienced moderately intensive childcare (part-time care before the age of 1½ years, followed by full-time care) reported lower levels of physical aggression and oppositional behavior compared to children who had received low-intensity child care. The effect on physical aggression, however, seemed to be specific to children from families with a low socio-economic status.

In a Swiss study involving a sample of 1,225 individuals, the impacts of NFECC on the development of children aged 7 to 20 years were examined [11]. This study compared five different types of NFECC (other family members, acquaintances/neighbours, daycare centers, individual day care providers, playgroups) across a broad spectrum of behavioral issues from the perspectives of parents, children, and teachers. The results were highly differentiated and complex, with the majority of coefficients not reaching significance. Depending on the type of NFECC, the informant, and the child's age, varied effects were observed. Depending on the type of care and social status of the family, a higher level of care was associated with more externalizing and internalizing behavior problems, at least up to the age of 11 years. This finding, however, was not consistent across all types of child care. While a higher level of care in daycare centers was associated with more externalizing and internalizing behavior problems, this association was found only for externalizing behavior problems when analyzing individual day care providers. The results furthermore varied based on the families’ socioeconomic background, because for children from socioeconomically disadvantaged families, attending a daycare center was associated with less externalizing but more internalizing behavior problems. In addition, for children from socioeconomically disadvantaged backgrounds, a longer stay in a daycare center was associated with higher substance use in adolescence. The authors however emphasized that causal conclusions cannot be drawn from their data because of the cross-sectional design of the study.

Within the field of economics, a research domain has emerged focusing on the consequences of NFECC, known as the "Economics of Child Care." In the United States, Heckman et al. [12] demonstrated that NFECC, particularly in the early years, is of considerable benefit to socially disadvantaged children. For instance, the rate of return was 1:17 for the HighScope Perry Preschool Program, indicating that for every U.S. dollar invested within the program, 17 U.S. dollars were later saved. A German study by the Bertelsmann Foundation [13] investigated the influence of NFECC on later school attendance and the anticipated lifetime income. The authors found that, for children in NFECC, the probability of later attending a German high school increased from 36 to 50%, with the relative gain being most pronounced in children with a lower socioeconomic background. The monetary economic benefit, measured by a child’s additional lifetime income in relation to the daycare costs, was estimated at 1:2.7.

The mechanisms of adverse effects of NFECC on children are receiving attention in stress research. It is presumed that NFECC is associated with increased stress levels in children recorded by daily cortisol measurements (e.g. [14]). The developmental risks associated with NFECC are primarily explored from psychoanalytic and cognitive theoretical frameworks [15].

The current state of research on NFECC therefore presents a complex and partly inconsistent picture. Findings on its positive and negative effects vary considerably, with studies in stress research generally indicating small but negative effects on children’s stress levels in both cross-sectional and longitudinal designs. At the same time, outcomes appear to differ depending on family background: children from socially disadvantaged families, including those with lower socioeconomic status, single-parent households, or migration and non-Western backgrounds, tend to benefit more from NFECC, whereas children from two-parent, middle-class families more often show negative effects. The quality of care emerges as a crucial factor, consistently showing positive impacts on child development, while evidence regarding the influence of daily childcare duration remains contradictory. Early entry into NFECC, particularly before the age of one, is more frequently associated with negative developmental outcomes. Overall, family characteristics – especially maternal sensitivity – are stronger predictors of later child development than the type of childcare itself. Notably, most existing studies are based in the United States and other European countries, raising questions about their applicability to the German care system [16]. Furthermore, there is limited evidence on long-term effects extending into young adulthood, a gap that the present longitudinal study seeks to address by focusing on the German context.

### The present study – research questions and hypotheses

In this study, the impact of NFECC on child development in young adulthood was examined (young adults’ age: *M* = 22 years at the 18-year follow up [FU18]). The central research questions of this study were as follows:

1. How is early childhood care *distributed* within and outside the family? Do parents of children in NFECC differ from those who look after their child themselves in terms of sociodemographic and psychological risk factors? It was assumed that children from high-risk families are more often in NFECC compared to children from low-risk families.
2. What are the *effects* of NFECC compared to parental care on the development of child mental health problems, protective factors, risk behaviors, and academic as well as vocational achievement in young adulthood? Due to inconsistent international research findings and a lack of relevant German studies, it is not possible to formulate a directional hypothesis.
3. Are the effects of NFECC in young adulthood *moderated* by the families’ socioeconomic background, migration background, parental status (e.g., one- vs. two-parent household) or child sex? Children with a low socioeconomic background, a migration background, and those raised in single-parent households were expected to benefit significantly more from NFECC, whereas sex was not anticipated to be a significant moderating variable.
4. Does the *age of entry* into non-familial care have a significant effect on child outcome (mental health problems, protective factors, risk behaviors, academic and vocational achievement)? It was assumed that an early age of entry is negatively associated with the development of mental health problems, protective factors, risk behaviors, and academic as well as vocational achievement in young adulthood.

### Excursus: childcare in Germany, specifically in Braunschweig

**Fig. 1:**
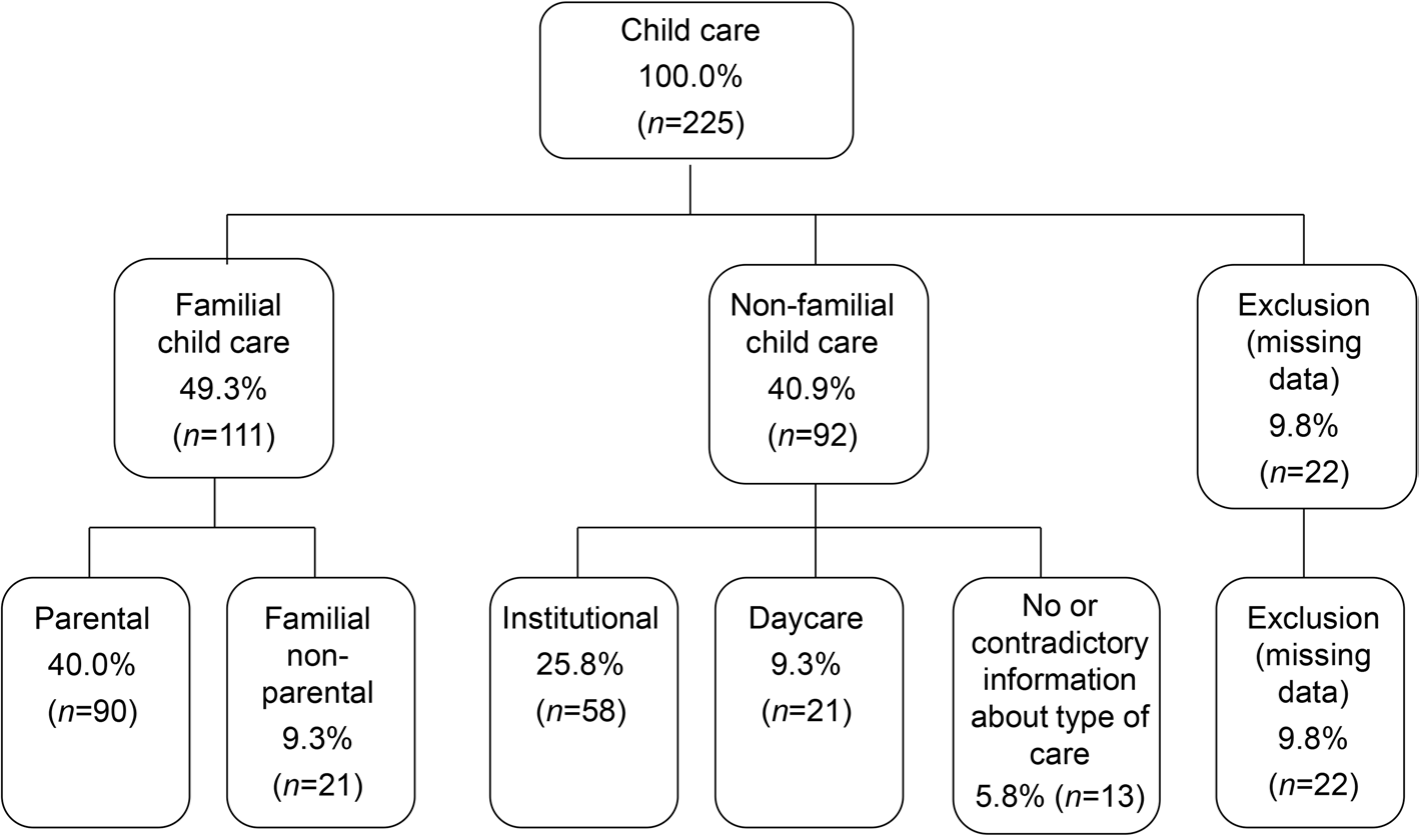
Flowchart of the individual types of child care

In Germany, childcare for children under age three (U3) is provided either within the family (primarily by parents or grandparents) or outside the family (non-familial), for example nurseries (institutional care) or by childminders (daycare) (see Fig. 1). At the time of our study, the staff-to-child ratio in nurseries was 1:7.5, with an average daily care time of four to five hours. Between the ages of three and six, children typically attended kindergartens, which generally offer full-day care with a staff-to-child ratio of 1:12.5.

In recent years, NFECC for children under three has increasingly become a focus of public and political attention in Germany and the care situation has mostly improved. Since 2013, there has been a legal entitlement to a daycare place for children aged two to three. For children in their first year, this entitlement is subject to conditions, such as the requirement that their parents are employed. In 2019 and 2023, two national laws came into force (“Gute-Kita-Gesetz/Good Daycare Law” and “KiTa-Qualitätsgesetz/Daycare Quality Law”) aimed at improving the quality of care, for example, by increasing the caregiver-to-child ratio (1:3,0 to 1:6.7) and recruiting and retaining qualified professionals. These measures were intended to contribute to reducing educational inequalities and improving the compatibility of family and career in Germany. The laws are currently being implemented in the country.

## Methods

The present study was conducted within the "Future Family" (FF) project, an 18-year longitudinal study. The FF project originally consisted of two studies: the FF-I study, a randomized controlled study, and the FF-II study, a non-controlled study. Both studies investigated the effectiveness of the Triple P—Positive Parenting Program (for further details see [17]). Within FF-III the 10-year follow-up (FU10; [17]) and within FF-IV the 18-year follow-up (FU18) of the two original samples were assessed. The primary focus of the FF project was to examine the development of child mental health problems, taking into account risk and protective factors in kindergarten age, adolescence, and young adulthood. Since data on NFECC were only collected in the FF-I study, the present study exclusively examined families from the FF-I sample (*N* = 280).

### Sample recruitment

All 33 daycare centers in Braunschweig (a midsize German city) were informed about the FF-I research project, and 23 expressed their interest in participating. Subsequently, 17 centers were randomly selected, and families from these centers were recruited in the years 2001/2002. The initial sample consisted of 280 families. At the first assessment (Pre, FF-I), the children had an average age of 4.1 years (*SD* = 1.0). Eighteen years later (FU18, FF-IV), 225 families still participated, resulting in a retention rate of 80%.

### Procedure

The data were collected using a combination of personal interviews with one parent and the young adults separately, along with standardized questionnaires. Due to the COVID-19 pandemic, most interviews were carried out by telephone. Prior to the assessments, all interviewers underwent an intensive training. Questionnaires were administered using traditional paper and pencil methods or online via SurveyMonkey (www.surveymonkey.de) for the FU18 assessment. The participating parent and young adults each received a financial compensation of € 50 for their involvement in the approximately 2.5-h interviews.

### Measures

The *sociodemographic characteristics*, including child's sex, family status, data on the educational level of the mother and father, monthly household income, and migration background, were collected during the Pre interview. A migration background for a child was considered when at least one parent did not possess German citizenship by birth [18]. The age of the children and their parents, along with data on the educational level and current vocational training or occupation of the young adults, were assessed during the FU18-interview. The socioeconomic status at FU18 was determined using the Scheuch-Winkler Index of the German KiGGS-Study (Wave 2) [19], with the child's index being computed as the mean of the mother's and father's indices.

Information on *early childhood care* up to the age of 3 years was retrospectively collected from mothers during the Pre interview. Mothers were asked by whom (parents, relatives, daycare providers, etc.) and to what extent (in hours) their child was cared for up to the age of 3 years and at what age the child entered daycare or kindergarten. Following the approach of Vandell et al. [20], children who received *less than ten hours* of non-familial care per week were categorized into the group of parentally cared-for children. Children receiving *ten or more hours* of non-parental care were assigned to the respective NFECC group. In cases of missing or contradictory information, these families were excluded from the following analyzes (see Fig. 1).

For the *assessment of mental health problems in young adulthood (FU18)*, the following instruments were used: (1) The German version of the Adult Self-Report for Ages 18–59 (ASR/18–59; [21]) was used to assess internalizing and externalizing behavior problems; t-scores; a higher score indicates more internalizing and externalizing behavior problems; (2) Symptoms of anxiety were assessed using the German version of the Generalized Anxiety Disorder Questionnaire (GAD-7; [22]; 7 items; score: 0–21; a higher score indicates more symptoms of anxiety); (3) Symptoms of depression were assessed using the German version of the Depression module of the Patient Health Questionnaire (PHQ-9; [23]; 9 Items; score: 0–27; a higher score indicates more symptoms of depression).

In addition, protective factors and risk behaviors were being investigated at FU18 using the German versions of the following standardized and validated questionnaires:

*Protective factors* were assessed at FU18 using the following measures: 1. Life Satisfaction Scale (LSS; [24]; 8 items from different areas of life; combined value of importance and satisfaction; score: −96 – 160; a higher score indicates more life satisfaction; 2. Positive Mental Health-Scale (PMH; [25]; 9 Items; score 9–36; a higher score indicates more positive mental health); 3. Social Support Questionnaire (SSQ; [26]; 14 Items; score 14–70; a higher score indicates more social support); 4. Abbreviated Dyadic Adjustment Scale (ADAS; [27]; 7 Items; score 0–36; a higher score indicates higher relationship quality); 5. Resilience Scale (RS-11; [28]; 11 Items; score 11–77; a higher score indicates higher resilience).

*Risk behaviors* were assessed at FU18 using the following measures: 1. Sexual Risk Behavior [29]; 11 Items; score 0–11; a higher score indicates more sexual risk behavior; 2. Lübeck Alcohol Screening Test (LAST; [30]; 7 Items; score 0–7; a higher score indicates more symptoms of alcohol dependence and abuse), 3. Cannabis Use Disorders Identification Test (CUDIT; [31]; distinguishing between cannabis use vs. non cannabis use; 4. Tobacco use was asked about during the personal interview, distinguishing between smokers and non-smokers.

### Determination of the risk index

In line with previous research on risk factors for mental health problems in children and adolescents [32–34], the following eight variables (each dichotomized) collected at Pre-assessment were selected to determine an individual risk index for the young adults: 1) low maternal education level (either a primary school diploma or no educational attainment and/or no completed vocational training); 2) raised in a single parent household; 3) increased psychopathology in mothers (elevated scores on at least one of the three subscales of the German version of the Depression Anxiety Stress Scales, DASS-42; [35]); 4) maternal substance use (alcohol consumption during pregnancy or smoking during pregnancy or while breastfeeding); 5) physical punishment by the mother ("no physical punishment" vs. "physical punishment"); 6) low socioeconomic status, as indicated by a low daycare social structure index, (which is based on factors such as unemployment rate, number of families receiving welfare, number of immigrants, and the quality of housing in the daycare center’s area; [36]); 7) monthly family income (< 2,500 German Mark); 8) early child mental health problems (CBCL 1½—5; [37], cut-off: at least one standard deviation above the sample mean).

Based on the Mannheim Children at Risk Study [32] and the BELLA Study [33], for each young adult, a cumulative risk index was calculated as an unweighted sum from the eight risk factors described above (range: 0 to 8).

### Statistical analyses

Not all participants provided complete questionnaire or interview data. In cases where entire scales were missing, the analyses were conducted without those participants, whereas in cases where only individual item responses were missing (up to a maximum of 15%), the missing values were imputed using the mean of the other items.

To investigate differences between parental and NFECC regarding sociodemographic and psychological risk factors of families (*Research Question 1*), *t*-tests and *χ2*-tests, along with effect sizes (*d*, φ-coefficient), were computed. For the examination of the effects of the type of care on child outcomes in young adulthood (*Research Question 2*), *χ*^*2*^-tests and analysis of covariance (ANCOVA) (with the individual risk index at Pre as a covariate) were conducted, again with corresponding effect sizes (η^2^, ɸ-coefficient). Additionally, logistic regressions were calculated, using the individual risk index at Pre as a control variable.

Moderator analyses were performed to assess potential moderating effects of socioeconomic status, migration background, single parent status and child sex on the relationship between type of care and outcomes (criteria: mental health problems, protective factors, risk behavior, educational level and professional attainment) in young adulthood (research question 3).

A moderation effect is identified by a significant interaction effect between the predictor and moderator. The PROCESS program for IBM SPSS Statistics by Hayes [38] was utilized for the computation of possible moderation effects. In the case of a significant interaction effect, slope analyses were conducted to examine how the change in the relationship between predictor and criterion was influenced by the moderator.

To investigate the influence of the age of entry into NFECC on the criteria (*Research Question 4*), univariate linear regressions were performed with the sub-sample of young adults who had been in non-familial care (*n* = 92).

In statistical analyses, *p*-values ≤ 0.05 are considered significant, *p*-values ≤ 0.01 very significant, and *p*-values ≤ 0.001 highly significant. Additionally, due to the exploratory nature of the study, *p*-values ≤ 0.10 are considered indicative of a trend toward significance. Since this study involves multiple testing, the Benjamini–Hochberg procedure was used to control the expected False Discovery Rate (FDR).

The interpretation of the effect sizes is based on the usual guidelines: *d* ≥ 0.20 small, *d* ≥ 0.50 medium, *d* ≥ 0.80 large effect; *ϕ/r/ß* > 0.10 small, *ϕ/r/ß* > 0.30 medium, *ϕ/r/ß* > 0.50 large effect; *η2* > 0.01 small, *η2* > 0.06 medium, η2 > 0.014 large effect.”

## Results

### Sample characteristics

At FU18, the young adults were on average 22.6 years old (*SD* = 1.1). Further details on sample characteristics are summarized in Table 1.

**Table 1:** Sample characteristics at the 18-year follow-up (*N* = 225)

Sample Characteristics	*M*	*SD*
Age of young adults (FU18)	22.6	1.1
Age of mothers (FU18)	54.1	4.6
Age of fathers (FU18)	56.5	4.8

	***N***	**%**
Sex of young adults (Pre):		
- Female	111	49.3
- Male	114	50.7
Family status (Pre):		
- Two-parent family	184	81.8
- Single mothers	40	17.8
- Single fathers	1	0.4
Migration background (Pre):		
- No migration background	201	89.3
- Migration background	24	10.7
Educational level of mothers (Pre):		
- Without school leaving certificate, 9 classes	16	7.1
- 10 classes	78	34.8
- A-Levels/High school	130	58.0
Highest school degree of fathers (Pre):		
- Without school leaving certificate, 9 classes	21	11.4
- 10 classes	47	25.6
- A-Levels/High school	116	63.0
Monthly household income (German Mark; DM) (Pre):		
- < 2.500	29	13.2
- 2.500 to < 6.000	117	53.4
- ≥ 6.000	73	33.3
Socioeconomic status (Pre):		
- Low status	7	3.1
- Middle status	67	29.8
- High status	149	66.2
Relationship (FU18)		
- currently in a relationship	99	45.4
- currently not in a relationship	119	54.6
Education level young adult (FU18)		
- No school leaving degree, 9 classes school leaving degree (Hauptschule)	15	6.7
- 10 classes school leaving degree (Realschule)	30	13.4
- 12/13 classes A-Levels	179	79.9
Vocational education (FU18):		
- No diploma/no training	31	14.0
- Currently undergoing apprenticeship or completed apprenticeship	58	26.1
- Currently attending university or university degree	113	59.9

#### Representativeness of the FU18 sample

A dropout analysis showed significant differences between young adults who participated and who dropped out: Dropouts were more likely to have grown up in single-parent households (*p* = 0.003), more often had a low socioeconomic background (mother's and father's education, both *p* < 0.001; lower monthly household income, *p* = 0.014), and had younger mothers (*p* = 0.029). The representativeness of the FU18-sample is therefore limited compared to the baseline (Pre) sample (see Table A1 in the Supplementary).

### Types of child care: descriptive results

Of the 225 children, 40.0% (*N* = 90) were in parental care, while 40.9% (*N* = 92) were in NFECC. Figure 1 illustrates the complete group distribution. Of the 90 children in parental care, 36 (40%) also received additional NFECC (M = 5.3 h per week; SD = 2.2; range: 1 to 9).

### Risk index: statistical parameters und correlations with the outcome criteria

The average number of risk factors was 1.82 (SD = 1.33; range: 1–7). 27.7% of young adults had more than 3 risk factors. There were significant correlations between the risk index and the level of education and vocational attainment. The detailed results can be found in Table A2 in the Supplementary.2.

**Table 2:** Differences between parental and Non-Familial Early Childhood Care (NFECC): child mental health problems, protective factors, risk behaviors, education level and vocational attainment in young adulthood (FU18), Analysis of Covariance (ANCOVA) with risk index and participation in Triple P at pre as covariates and effect sizes

Criterion		*N*	*M*	*SD*		*F*	*p*^*A*^	η^2 A^	η^2^_*Covariate*_ Risiko	η^2^_*Covariate*_ Triple P
Mental Health Problems										
ASR Internalizing behavior problems	parental	83	54.8	11.0		0.03	.860	.000	.000	.000
	NFECC	87	54.5	13.4						
ASR Externalizing behavior problems	parental	83	49.7	8.6		2.01	.158	.012^#^	.001	.018^#^
	NFECC	87	51.8	9.8						
Anxiety symptoms (GAD-7)	parental	83	4.5	3.9		0.17	.677	.001	.001	.004
	NFECC	88	4.8	4.2						
Depressive symptoms (PHQ-9)	parental	83	5.7	4.6		0.66	.417	.004	.004	,000
	NFECC	88	6.3	5.3						
Protective Factors										
General Life satisfaction (LSS)	parental	83	54.6	30.0		0.02	.893	.000	.003	.000
	NFECC	87	55.8	35.3						
Positive mental health (PHM)	parental	80	28.4	5.9		0.51	.476	.003	.014^#^	.003
	NFECC	88	28.8	6.1						
Perceived social support (SSQ)	parental	83	62.8	7.6		0.23	.633	.001	.011^#^	.004
	NFECC	87	61.9	8.7						
Relationship quality (ADAS)	parental	33	26.5	4.0		4.41	.039*	.056^#^	.017^#^	.000
	NFECC	46	24.2	5.5						
Resilience (RS-11)	parental	83	57.3	8.6		0.00	.996	.000	.002	.003
	NFECC	87	57.1	11.6						
Risk behaviors										
Risky sexual behavior	parental	80	1.8	2.2		6.24	.013*	.037^#^	.000	.001
	NFECC	88	2.7	2.3						
Alcohol abuse (LAST)	parental	80	0.7	1.0		6.62	.011*	.039^#^	.011	.002
	NFECC	87	1.2	1.4						
Cannabis use (CUDIT)^1^		Cannabis abuse		No Cannabis abuse						
		*N*	%	*N*		%	χ^2^-Test	*p*	ɸ^7^	
	parental	35	43.7	45		56.3	9.59	.002**	.238^#^	
	NFECC	60	67.4	29		32.6				
Tobacco use^2^		Tobacco use		No tobacco use						
		*N*	%	*N*		%	χ^2^-Test	*p*	ɸ^7^	
	parental	28	33.7	55		66.3	7.90	.005**	.214^#^	
	NFECC	49	55.1	40		44.9				
Education level and vocational attainment										
Education level^3^		None/9 classes		10 classes		12/13 classes A-Levels				
		*N*	%	*N*	%	*N*	%	χ^2^-Test^4^	*p*	ɸ^7^
	parental	7	7.8	13	14.4	70	77.8	1.39	.239	.088
	NFECC	6	6.6	8	8.8	77	84.6			
Vocational attainment^5^		None		Apprenti-ceship		University				
		*N*	%	*N*	%	*N*	%	χ^2^-Test^6^	*p*	ɸ^7^
	parental	13	14.6	30	33.7	46	51.7	5.799	.016*	.179^#^
	NFECC	13	14.3	15	16.5	63	69.2			

^*A*^ after controlling for the risk index and participation in Triple P

^1^ after controlling for the risk index *p*=.002**, *OR*=2.84^##^; Risk index *p*=.154, *OR*=1.21; Triple P *p*=.086^+^, *OR*=1.75^#^ (logistic regression)

^2^ after controlling for the risk index *p*=.006**, *OR*=2.45^#^; Risk index *p*=.803, *OR*=1.03; Triple P *p*=.053^+^, *OR*=1.86^#^ (logistic regression)

^3^ after controlling for the risk index *p*=.109, *OR*=1.92^#^; Risk index *p*=.014*, *OR*=0.69^#^; Triple P *p*=.526, *OR*=1.28 (logistic regression)

^4^ Categories "None/9 classes" and "10 classes" combined

^5^ after controlling for the risk index *p*=.005**, *OR*=2.56^##^; Risk index *p*=.003**, OR=0.67; Triple P *p*=.703, *OR*=1.13 (logistic regression)

^6^ Categories "No Degree" and "Apprenticeship" combined

^7^ ɸ indicates effect size for χ^2^-Test

^+^*p*<.10, *p<.05, **p<.01, ***p<.001; ^#^small effect size, ^##^ middle effect size, ^###^ high effect size

### Comparison of parental child care and NFECC: sociodemographic data and risk index (Pre) (Research Question 1)

When comparing parental care and NFECC, significant differences were found based on the individual risk index (*p* = 0.012, *d* = −0.35, *T* = −2.55, M_familial_ = 1.64, M_NFECC_ = 2.13). The higher the risk index at Pre, the more frequently children were in NFECC. Significant differences were furthermore found regarding specific risk factors: Children with both a low and high socioeconomic status tended to be in NFECC more frequently compared to children with a middle socioeconomic status (mothers' education: *p* < 0.05; monthly household income: *p* < 0.01). In addition, significantly higher rates of NFECC were observed in single-parent households compared to two-parent households (*p* < 0.05). Additional analyses showed that families with only one child tended to opt for NFECC significantly more often than families with at least two children (*p* < 0.05). No differences were found based on child's sex (*p* = 0.630) or migration background (*p* = 0.620).

There was a significant correlation between the individual risk index and age of entry into NFECC (*r* = −0.39, *p* < 0.001): Children with a higher risk index entered NFECC earlier. Due to the significant associations, the individual risk index was included as a control variable in the analyses of Hypotheses 2 and 4.

### Differences between parental care and NFECC: child mental health problems, protective factors, risk behaviors, and academic as well as vocational achievements in young adulthood (Research Question 2)

The results of the analysis of covariance (ANCOVA) and the χ^2^ tests with individual risk index as a control variable are shown in Table 2..

In terms of *mental health problems* (ASR), no significant differences were found between parental care and NFECC after controlling for the individual risk index und Tripl P. However, a descriptive examination of the means and effect sizes revealed that young adults in NFECC consistently exhibited more externalizing problems compared to young adults in parental care (η2 = 0.012).

Significant differences in *protective factors* were only found regarding intimate couple relationship satisfaction, indicating that young adults in NFECC were less satisfied with their intimate relationship (*p* = 0.039, η2 = 0.056).

Regarding *risk behaviors*, significant differences were found for risky sexual behavior (*p* = 0.013, η2 = 0.037), alcohol abuse (*p* = 0.011, η2 = 0.039), cannabis abuse (*p* = 0.002, ɸ = 0.238), and tobacco use (*p* = 0.005, ɸ = 0.214), with small effect sizes. Young adults who had been in NFECC were significantly more likely to engage in these risk behaviors compared to young adults who had been in familial care.

In terms of *vocational attainment*, there was a significant difference (*p* = 0.016) with a small effect size (ɸ = 0.179), even after controlling for the individual risk index and Triple P. Contrary to expectations, young adults in NFECC more frequently pursued higher education or already had a degree (e.g., a university degree).

After the Benjamini–Hochberg correction, the risk factors of risky sexual behaviour, alcohol abuse, cannabis abuse, tobacco use, and vocational attainment were still significant (see Table A3 in the Supplementary).

**Table 3:** Results of Moderator Analyses at FU18

	Moderator Analyses							
	Migration background		Socioeconomic status		Single parents		Sex	
Criterion	*p*	*η*^*2*^	*p*	*η*^*2*^	*p*	*η*^*2*^	*p*	*η*^*2*^
Mental health problems								
ASR Internalizing	.082^+^	**.018**^#^	.261	**.016**^#^	.241	.008	.516	.003
ASR Externalizing	.152	**.012**^#^	.480	.009	.394	.004	.739	.001
Anxiety symptoms (GAD-7)	.102	**.016**^#^	.187	**.020**^#^	.920	.000	.237	.008
Depressive Symptoms (PHQ-9)	.008**	**.041**^#^	.240	**.017**^#^	.858	.000	.733	.001
Protective factors								
Life satisfaction (LSS)	.011*	**.038**^#^	.026*	**.044**^#^	.755	.001	.659	.001
Positive mental health (PHM)	.034*	**.027**^#^	.123	**.026**^#^	.233	.009	.552	.002
Social support (SSQ)	.334	.006	.445	**.010**^#^	.477	.003	.164	**.012**^#^
Relationship quality (ADAS)	.459	.007	.904	.000	.254	**.017**^#^	.797	.001
Resilience (RS-11)	.041*	**.025**^#^	.344	**.013**^#^	.456	.003	.869	.000
Risk behaviors								
Sexual risk behavior	.095^+^	**.017**^#^	.909	.001	.243	.008	.624	.001
Alcohol abuse (LAST)	.353	.005	.001**	**.085**^##^	.735	.001	.975	.000
Cannabis use (CUDIT)	.224	**.032**^#^	.904	.005	.995	.000	.792	.000
Tobacco use	.046*	**.024**^#^	.321	**.014**^#^	.598	.002	.430	.004
Educational level and vocational attainment								
Educational level	.135	**.013**^#^	.170	**.020**^#^	.840	.000	.218	.009
Vocational attainment	.550	.002	.191	**.019**^#^	.811	.000	.309	.006

^+^*p* <.10, *p <.05, **p <.01, ***p <.001; ^#^small effect size, ^##^ middle effect size, ^###^ high effect size, bold: significant or at least small effects

### Moderator analyses (Research Question 3)

To examine potential moderating effects of migration background, socioeconomic status, family status, and sex on child outcomes in young adulthood (mental health problems, protective factors, risk behaviors, education level, and vocational attainment), moderator analyses were conducted. The results can be found in Table 3 in the electronic supplement material. Contrary to expectations, family status (one-parent vs. two-parent household) did not have a moderating effect on the impact of NFECC on child outcome; none of the interaction effects were significant. Similarly, sex did not have a moderating effect either, aligning with our expectations. In contrast, the analyses yielded some significant interaction effects for migration background and socioeconomic status, while others were not significant, but resulted in at least small effect sizes.

#### Migration background

**Fig. 2:**
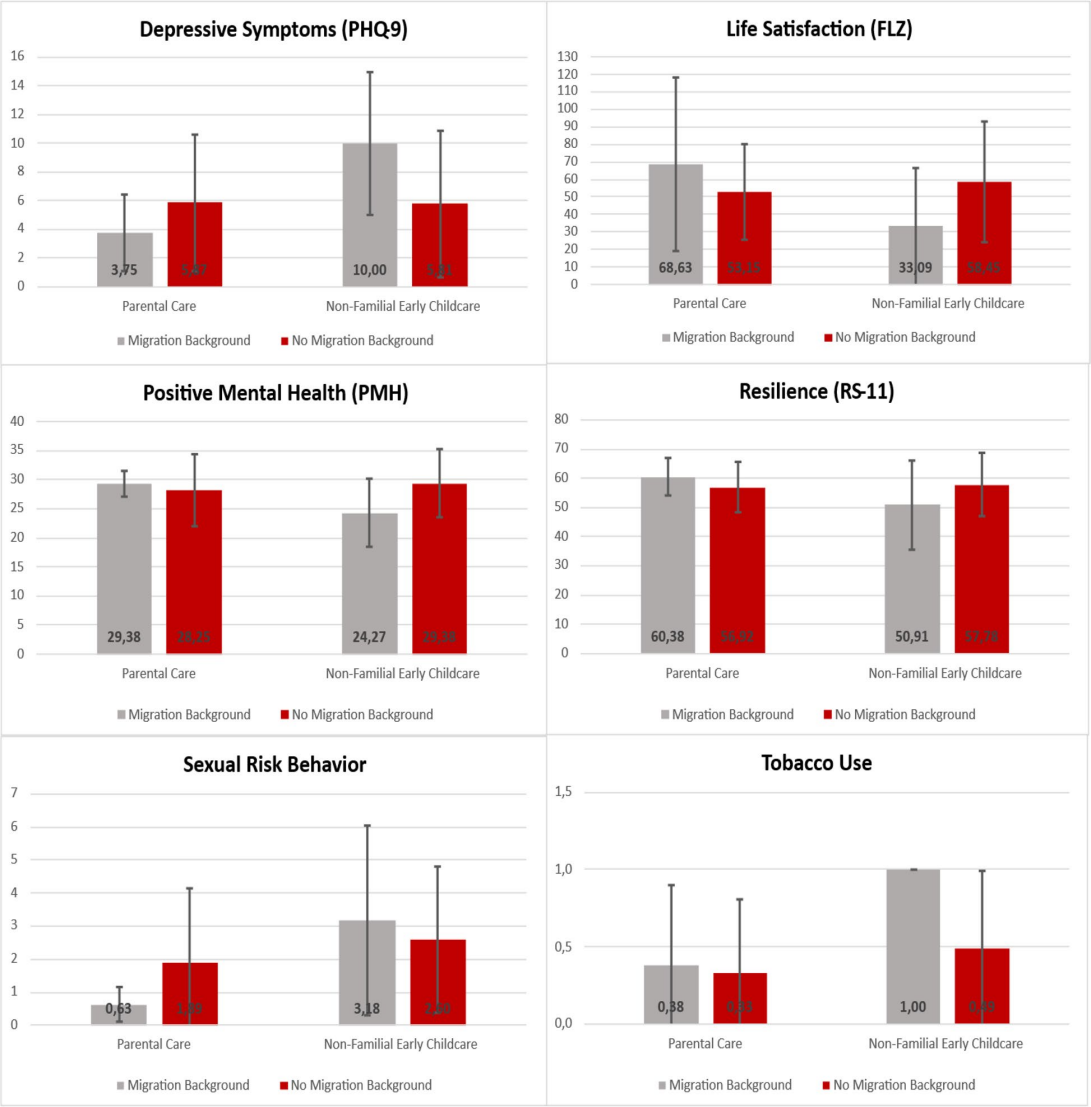
Results of the slope analyses of significant interaction effects in moderator analyses with the predictor Non-Familial Early Childcare (NFECC) and the moderator migration background

For the following child outcomes, a significant moderating effect of migration background was found: Depressive symptoms (PHQ-9; *p* = 0.008, η2 = 0.041), general life satisfaction (LSS; *p* = 0.011, η2 = 0.038), positive mental health (PHM; *p* = 0.034, η2 = 0.027), resilience (RS-11; *p* = 0.041, η2 = 0.025), and tobacco use (*p* = 0.046, η2 = 0.024) (see Table 3). To interpret the significant interaction effects, e.g., to determine how the change in the association between NFECC and the outcome is affected by migration background, slope analyses were conducted. The slope analyzes on the migration background were exploratory, as they were based on a very small sample of migrants (*N* = 19; parental care: *N* = 8, NFECC: *N* = 11). The results of the slope analyses are summarized in Table A4 in the Supplementary and Fig. 2. It was found that young adults without a migration background did not differ depending on whether they were in parental care or in NFECC. No outcome showed a significant effect. Young adults in parental care, however, tended to report less sexual risk behavior, and less frequent tabaco use. In contrast, for young adults with a migration background the analyses consistently yielded significant (or at least trending) results, except for tobacco use, indicating that NFECC leads to less favourable child outcomes. Children with a migration background who had been in NFECC reported significantly more symptoms of depression, a significantly lower general life satisfaction and resilience, reported to engage more frequently in risky sexual behavior, and overall had poorer mental health outcomes in young adulthood.

**Table 4:** Results of the univariate linear regression models in the subgroup of Non-Familial Early Childhood Care (NFECC) children with age of entry into non-familial care as a predictor and the individual risk index and participation in triple P as control variables

Criterion	*B*^*1*^	ß^1^	*t*	*p*	*r*	*corr. R*^*2*^
Mental Health Problems						
ASR Internalizing (*N* = 87)						
- Age of entry	−0.28	-.225^#^	−1.98	.051^+^	.221	.015
- Risk index	−0.10	-.010	−0.09	.927		
- Participation in Triple P	0.10	.004	0.03	.973		
ASR Externalizing (*N* = 87)						
- Age of entry	−0.15	-.167^#^	−1.46	.147	.186	.000
- Risk index	−0.01	.-.001	−0.01	.995		
- Participation in Triple P	−1.11	-.057	−0.52	.604		
Anxiety symptoms – GAD-7 (*N* = 88)						
- Age of entry	−0.08	-.205^#^	−1.80	.076^+^	.220	.014
- Risk index	−0.11	-.036	−0.31	.754		
- Participation in Triple P	−0,65	-.077	−0.72	.475		
Depressive symptoms—PHQ-9 (*N* = 88)						
- Age of entry	−0.11	-.222^#^	−1.95	.054^+^	.212	.011
- Risk index	−0.44	-.111^#^	−0.98	.331		
- Participation in Triple P	0.03	.002	0.02	.983		
Protective factors						
Life satisfaction—LSS (*N* = 87)						
- Age of entry	0.35	.107^#^	0.93	.331	.140	.016
- Risk index	2.98	.113^#^	0.98	.355		
- Participation in Triple P	3.72	.053	0.48	633		
Positive mental health—PMH (*N* = 88)						
- Age of entry	0.12	.218^#^	1.93	.057^+^	.254	.031
- Risk index	−0.24	-.051	−0.46	.649		
- Participation in Triple P	0.66	.054	0,51	.613		
Perceived Social support—SSQ (*N* = 87)						
- Age of entry	0.20	.247^#^	2.21	.030*	.279^+^	.044
- Risk index	−0.13	-.019	−0.17	.864		
- Participation in Triple P	1.38	.079	0,74	.463		
Relationship quality—ADAS (*N* = 46)						
- Age of entry	0.24	.183^#^	2.43	.019*	.353	.062
- Risk index	0.85	.406^##^	1.16	.252		
- Participation in Triple P	−1.75	-.161^#^	−1.04	.305		
Resilience—RS-11 (*N* = 87)						
- Age of entry	0.24	.224^#^	1.97	.052^+^	.225	.016
- Risk index	0.37	.042	0.38	.709		
- Participation in Triple P	0.98	.043	0.39	.696		
Risk behaviors						
Risky sexual behavior (N = 88)						
- Age of entry	−0.04	-.110^#^	−0.96	.340	.185	.000
- Risk index	−0.12	-.066	−0.58	.561		
- Participation in Triple P	−0.65	-.142	−1.30	.196		
Alcohol abuse—LAST (*N* = 87)						
- Age of entry	0.01	.036	0.37	.845	.036	.025
- Risk index	0.02	.019^#^	0.20	.712		
- Participation in Triple P	−0.01	-.003	−0.03	.976		
Cannabis abuse – CUDIT (*N* = 88)						
- Age of entry	−0.00	-.043	−0.37	.713	.089	.028
- Risk index	0.02	.042	0.36	.718		
- Participation in Triple P	0.06	.068	0.61	.541		
Tobacco use (*N* = 88)						
- Age of entry	−0.01	-.106^#^	−0.92	.358	.172	.005
- Risk index	−0.01	-.018	−0.16	.873		
- Participation in Triple P	−0.13	-.126^#^	−1.16	.251		
Education level and vocational attainment						
Education level (*N* = 90)						
- Age of entry	0.01	.209^#^	1.88	.063^#^	.296	.056*
- Risk index	−0.08	-.144^#^	−1.31	.194		
- Participation in Triple P	0,03	^.^017	0.17	.868		
Vocational attainment (*N* = 90)						
- Age of entry	0.00	.015	0.13	.894	.233	.021
- Risk index	−0.14	-.201^#^	–1.79	.077^#^		
- Participation in Triple P	0.15	^.^083	0.78	.438		

^*1*^ The age of entry was recorded in months. Positive B or ß values ​​indicate: the later the age of entry, i.e., the older the children were, the higher the criterion score; negative B or ß values ​indicate​: the later the age of entry, i.e., the older the children were, the lower the criterion scores

^+^*p* <.10, *p <.05, **p < 01, ***p <.001, ^#^small effect size, ^##^ middle effect size

After the Benjamini–Hochberg correction, none of the effects were significant (see Table A5 in the Supplementary). The reported non-corrected results should therefore be interpreted with caution.

#### Socioeconomic status

For the following criteria, a significant moderating effect of the socioeconomic status was found: General life satisfaction (LSS; *p* = 0.026, η2 = 0.044) and alcohol abuse (LAST; *p* = 0.001, η2 = 0.085) (see Table A4 in the Supplementary). The subsequent slope analyses only yielded a significant effect for alcohol abuse. Both the middle and high socioeconomic status corresponded to lower alcohol abuse/dependence in parental care compared to NFECC, with the effect being significant only in the middle class (see Table A6 in the Supplementary).

After the Benjamini–Hochberg correction, only alcohol abuse remained significant (see Table A7 in Supplementary).

### Age of entry into non-familial early childhood care (Research Question 4)

To examine the effect of the age of entry into NFECC, univariate linear regressions were conducted with the subsample of NFECC children. Since age of entry significantly correlated with the individual risk index (Pre) (*r* = −0.35, *p* = 0.004), the risk index was considered as a control variable in the regression analyses. The results are displayed in Table 4.

Negative associations with small to medium effect sizes were predominantly observed between age of entry and *child mental health problems* in young adulthood: The earlier children were placed in NFECC, the more internalizing behavior problems (ASR-Int; *p* = 0.048, ß = −0.22), symptoms of anxiety (GAD-7; *p* = 0.060, ß = −0.21), and depressive symptoms (PHQ-9; *p* = 0.052, *ß* = −0.22) were reported in young adulthood. No significant impact was found for the individual risk index. The explained variance of the models was low.

Significant associations were also found for *protective factors*: An earlier age of entry lead to lower positive mental health (PHM; *p* = 0.047, ß = 0.23), less perceived social support (SSQ; *p* = 0.022, ß = 0.26), lower relationship quality (ADAS; *p* = 0.033, ß = 0.34), and lower resilience (RS-11; *p* = 0.044, ß = 0.23) in young adults. Again, the individual risk index had no significant impact, and the explained variance of the models was low.

The four examined risk behaviors showed no significant association with age of entry to NFECC. We furthermore found a significant association with a small effect size between age of entry and education level (*p* = 0.046, ß = 0.22). The later the entry of age, the higher the education level.

After the Benjamini–Hochberg correction, none of the effects were significant (see Table A8 in the Supplementary). The reported non-corrected results should therefore be interpreted with caution.

## Discussion

The aim of this study was to examine the positive and negative effects of NFECC on the development of children in young adulthood. In particular, we focused on mental health problems and risk behaviors. National as well as international studies report inconsistent findings so far.

In the following discussion of the results, it should be noted that families with low socioeconomic status were underrepresented in this study. Furthermore, the proportion of two-parent households was higher, and the proportion of families with a migration background was lower compared to national data. This may explain why negative effects of NFECC predominate in this study, and the results are most likely to be applicable to relatively privileged families (see Limitation 1 below).

First of all, the effect sizes found were predominantly small. Nevertheless, differences between parental care and NFECC were significant in 40% of the outcomes analyzed (even after controlling for early childhood risk factors), which is in line with previous international research (e.g. [7, 20, 39]). Given the nature of longitudinal studies over 18-years, it is not expected to observe large effects over such a long period of time. As time progresses, additional influencing factors become increasingly relevant (e.g., parental separation, peer influences), resulting in effects that may not be detected over a short period of time.

In detail, both in the parent-report as well as in the self-report, there were only minor differences in mental health problems between young adults who had been in NFECC compared to familial care. The most pronounced differences were found regarding externalizing behavior problems (e.g., aggressive behavior), to the disadvantage of the NFECC children. NFECC children furthermore consistently exhibited significantly higher rates of risky substance use (alcohol, tobacco, and cannabis use) and risky sexual behavior. Young adults who had been in familial care reported lower satisfaction with their intimate relationship compared to NFECC children. Regarding the educational level, no significant differences were found; contrary to expectations, the young adults who had been in NFECC were even slightly more likely to have a higher education level. In terms of vocational attainment, the effects were significant even after controlling for the individual risk index. Young adults with NFECC were more likely to be studying or already had a university degree. The predominantly negative outcomes are likely attributable to the low proportion of families from lower socioeconomic backgrounds in the sample.

The question arises as to which subgroups benefit from NFECC, and whether there are certain variables that moderate the association. Previous findings suggesting that the socioeconomic status acts as a moderator on the relationship between the type of care and mental health problems or certain risk behaviors, were not confirmed in the present study. Regarding life satisfaction and problematic alcohol consumption (abuse/dependence), significant interactions were found; however, subsequent slope analyses either indicated no significant differences (life satisfaction) or significant differences but convergent patterns (alcohol abuse).

Particularly, results from economic studies have shown that individuals from lower socioeconomic backgrounds benefit from NFECC (e.g. [12, 13]). A likely explanation for the non-significant findings in the study at hand might be the low proportion of families with a low socioeconomic status in the sample. Additionally, in this study all NFECC programs were universal and not aimed at specific target groups [7].

Apart from these results, significant effects of the individual risk index on certain outcome variables were found, particularly on protective factors, risk behaviors, and vocational attainment. The cumulative occurrence of risk factors in families is a significant predictor of mental health problems in young adulthood, which is in line with previous findings (e.g. [40]).

Moderator analyses were calculated in order to obtain indications of the different effects of familial care and NFECC.

The family status (two-parent families vs. single parents) did not emerge as a relevant moderator. There was no significant interaction for any of the examined dependent variables. This result is surprising in light of predominantly reported positive effects of NFECC among socially disadvantaged groups, including single-parent households [7].

As expected, there were no differences between young women and young men. They equally benefited from familial care or NFECC. In contrast, the migration background emerged as a significant moderator of mental health problems in young adults. When interpreting these findings, it should be noted, however, that they are based on a very small sample of migrants (*N* = 19, see above). The slope analyses indicate that there is no difference among young adults without a migration background as to whether they were cared for by their parents or NFECC.

In contrast, young adults with a migration background in NFECC correspond to less favorable values. They report significantly more depressive symptoms, lower life satisfaction, and a lower resilience, engage more frequently in risky sexual behavior, and overall, their mental health is poorer. Contrary to our expectations, NFECC appears to have long-term negative effects in this subgroup. Additional descriptive analyses showed that this negative effect was exclusively attributable to young adults with a one-sided migration background, individuals for whom it can be assumed that they exhibit a higher degree of integration compared to children whose parents both have a migration background. Given the small number of cases and the multiple testing in this study, replication with larger and representative samples is essential before far-reaching conclusions can be drawn about NFECC and migration background.

Perhaps, and this interpretation is certainly very speculative, young adults with a one-sided migration background are uncertain about which culture they belong to, making it harder for them to develop a clear identity. Recent societal discussions on regarding migration and integration policies in Germany may have contributed to increasing insecurities in developing one’s (cultural) identity. A problematic identity development may manifest as psychological distress and behavioral problems. Adolescents and young adults, especially those who have not (yet) formed a cultural identity, may represent a particular risk group in this context. Due to the small sample size of young adults with a migration background in the current sample, however, the results should be interpreted with caution. In their Swiss study Averdijk et al. [11] arrived at a comparable conclusion based on their results. Overall, these findings demonstrate how complex and nuanced the associations become when conducting differentiated analyses, considering multiple characteristics, perspectives, and subgroups.

Several studies already found that an early entry into NFECC institutional care has negative effects on child outcomes (e.g. [39]). This finding was confirmed in the current study. The earlier children entered NFECC, the more pronounced were the mental health problems in young adulthood. Given the fact that over 18 years have passed since the start of childcare, these effects are remarkable. When interpreting the results, however, it must be noted that after applying the Benjamini–Hochberg correction, there are no longer any significant effects and the effect sizes are small overall. It should also be noted that these findings are based only on the NFECC subgroup (*N* = 92), which further limits statistical power und precision. Further research with representative samples is needed, particularly regarding the age of entry and the quality of NFECC.”

### Limitations

Several limitations must be considered when interpreting the results. First, the generalizability of this study is limited due to the underrepresentation of families from lower socioeconomic backgrounds (1%; national average: 20%, [19]). The percentage of young adults (20 to 24 years) with a migration background is also lower than the national average (11% vs. 34%, [41]), as is the percentage of single-parent households (18% vs. national average: 23%, [42]). Second, although prior research highlights the importance of childcare quality (e.g., [20], also see [8]), this factor was not assessed and therefore could not be included in the analyses. Third, many children experienced multiple and overlapping forms of care with varying weekly hours, but this complexity was simplified in the present study by focusing only on the primary type of childcare using a 10-h threshold, suggesting that future research should more precisely account for individual care histories. Additionally, the categorization of NFECC groups relied on retrospective maternal reports, which may be subject to memory inaccuracies. Fourth, while the study’s 18-year longitudinal design is a clear strength, it also poses challenges, as the German childcare system has evolved over time, limiting direct comparability with today’s care situation. Fifth, because families self-selected childcare arrangements rather than being randomly assigned, internal validity is constrained, although this was partially addressed by controlling for family risk factors related to child mental health. The analyses involved multiple variables, increasing the risk of alpha error accumulation; although corrections using the Benjamini–Hochberg procedure were applied, the findings should still be interpreted with caution, particularly given the inclusion of exploratory *p*-values (≤ 0.10) and small effect sizes. Finally, despite a high retention rate suggesting minimal impact of the COVID-19 pandemic on participation, broader evidence indicates increased mental health problems during this period [43], although exploratory analyses in this study suggest that these increases were not associated with the type of childcare (e.g., the perceived impairment). Due to these limitations, the results should be considered exploratory and should be verified in future representative studies.

## Conclusions

Future research should investigate larger, representative samples and focus on examining moderating factors. In addition to the characteristics examined in the study at hand, particular attention should be given to the quality of NFECC, including the qualifications of care providers. Intrafamilial factors such as parental sensitivity and the number of siblings may also play a crucial role and warrant further investigation. Policymakers advocating for the expansion of NFECC facilities should not only consider the quantity but also emphasize the quality of these care facilities. Based on the results of the present study and the current state of research, parents should carefully consider the child's age of entry, the quality of care, and their own social situation when deciding for NFECC. Regardless of the decision made, the well-being of the child should always be the primary concern.

## Supplementary Information

Supplementary Material 1.

## Data Availability

The datasets generated and analyzed within the study at hand are not publicly available as they contain sensitive data. Furthermore, it is a longitudinal study with several assessment points. The data could possibly be used to draw conclusions about individuals. The questionnaires used can be found in the corresponding references.

## Abbreviations

NFECC: Non-familial early childhood care
FF: Future Family
FU: Follow-up

## References

[1] European Commission. Key data on early childhood education and care in Europe – 2019 Edition. Eurydice Report. Luxembourg: Publications Office of the European Union; 2019. https://www.dji.de/fileadmin/user_upload/icec/reports/Eurydice_Key-data_Europe_2019.pdf.

[2] Flisi S, Blasko Z, Stepanova E. Indicators for early childhood education and care. Reconsidering some aspects of the Barcelona target for younger children. Publications Office of the European Union, Luxembourg; 2022. 10.2760/44338, JRC130350.

[3] OECD. Enrolment in childcare and pre-school. OECD Family Database; 2023. https://www.oecd.org/els/soc/PF3_2_Enrolment_childcare_preschool.pdf.

[4] Statistische Ämter des Bundes und der Länder (2023). Kindertagesbetreuung regional: Karten zur Kindertagesbetreuung in Deutschland; 2023. https://www.statistikportal.de/de/karten/kindertagesbetreuung-regional. Accessed 08.02.2024.

[5] Jacob JI. The socio-emotional effects of non-maternal childcare on children in the USA: a critical review of recent studies. Early Child Dev Care. 2009;2009(179):559–70. 10.1080/03004430701292988.

[6] Lucas-Thompson RG, Goldberg WA, Prause J. Maternal work early in the lives of children and its distal associations with achievement and behavior problems: a meta-analysis. Psychol Bull. 2010;136:915–42. 10.1037/a0020875. 20919797 10.1037/a0020875

[7] Van Huizen T, Plantenga J. Do children benefit from universal early childhood education and care? A meta-analysis of evidence from natural experiments. Econ Educ Rev. 2018;66:206–22. 10.1016/j.econedurev.2018.08.001.

[8] Zemp M, Bodenmann G, Zimmermann P. Außerfamiliäre Betreuung von Kleinkindern. Wiesbaden: Springer; 2018.

[9] Linberg A, Burghardt L, Freund JD, Weinert S. Differential effect of duration of early childcare under the age of three on socio-emotional outcomes. Early Child Dev Care. 2019;190:2505–19. 10.1080/03004430.2019.1588891.

[10] Orri M, Tremblay RE, Japel C, Boivin M, Vitaro F, Losier T, et al. Early childhood child care and disruptive behavior problems during adolescence: a 17-year population-based propensity score study. J Child Psychol Psychiatry. 2019;60:1174–82. 10.1111/jcpp.13065. 31021429 10.1111/jcpp.13065

[11] Averdijk M, Ribeaud D, Eisner M. External childcare and socio-behavioral development in Switzerland: long-term relations from childhood into young adulthood. PLoS ONE. 2022;17(3):e0263571. 10.1371/journal.pone.0263571. 35263329 10.1371/journal.pone.0263571 PMC8906621

[12] Heckman JJ, Moon SH, Pinto R, Savelyev PA, Yavitz A. The rate of return to the HighScope Perry Preschool Program. J Public Econ. 2010;94:114–28. 10.1016/j.jpubeco.2009.11.001. 21804653 10.1016/j.jpubeco.2009.11.001 PMC3145373

[13] Fritschi T, Oesch T. Volkswirtschaftlicher Nutzen von frühkindlicher Bildung in Deutschland. Eine ökonomische Bewertung langfristiger Bildungseffekte bei Krippenkindern. Gütersloh: Bertelsmann Stiftung; 2008.

[14] Vermeer HJ, Groeneveld MG. Children’s physiological responses to childcare. Curr Opin Psychol. 2017;15:201–6. 10.1016/j.copsyc.2017.03.006. 28813263 10.1016/j.copsyc.2017.03.006

[15] Butzmann E. Entwicklungsrisiken bei früher Krippenbetreuung aus psychoanalytischer und kognitionstheoretischer Sicht. In: Sulz S, Walter A, Sedlacek F, editors. Schadet die Kinderkrippe meinem Kind? Worauf Eltern und Erzieherinnen achten und was sie tun können. München: Cip-Medien; 2018. p. 95–108.

[16] Zmyj N, Schölmerich A. Förderung von Kleinkindern in der Tagesbetreuung. In W. Schneider W, Lindenberger U. editors. Entwicklungspsychologie. 7 rd. ed. Weinheim: Beltz; 2012. p. 581–592.

[17] Hahlweg K, Schulz W. Universelle Prävention kindlicher Verhaltensstörungen durch Elterntrainings. Z Klin Psychol Psychother. 2018;47:1–15. 10.1026/1616-3443/a000462.

[18] Statistisches Bundesamt. Bevölkerung und Erwerbstätigkeit. Bevölkerung mit Migrationshintergrund - Ergebnisse des Mikrozensus 2015; 2016. https://www.statistischebibliothek.de/mir/servlets/MCRFileNodeServlet/DEHeft_derivate_00037315/2010220157004_korr21032017.pdf. Accessed 08.02.2024.

[19] Lampert T, Hoebel J, Kuntz B, Müters S, Kroll LE. Socioeconomic status and subjective social status measurement in KiGGS Wave 2. J Health Monit. 2018;3:108–25. 10.17886/RKI-GBE-2018-033. 35586179 10.17886/RKI-GBE-2018-033 PMC8848848

[20] Vandell DL, Belsky J, Burchinal M, Vandergrift N, Steinberg M. Do effects of early child care extend to age 15 years? Results from the NICHD study of early child care and youth development. Child Dev. 2010;81:737–56. 10.1111/j.1467-8624.2010.01431.x. 20573102 10.1111/j.1467-8624.2010.01431.x PMC2938040

[21] Achenbach T. Fragebögen zur Erfassung psychischer Probleme bei Erwachsenen (ASR/18-59 und ABCL/18-59). Deutschsprachige Fassungen des Adult Self-Report for Ages 18-59 und der Adult Behavior Checklist for Ages 18-59. Göttingen: Hogrefe; 2014.

[22] Spitzer RL, Kroenke K, Williams JB, Löwe B. A brief measure for assessing generalized anxiety disorder: the GAD-7. Arch Intern Med. 2006;166:1092–7. 10.1001/archinte.166.10.1092. 16717171 10.1001/archinte.166.10.1092

[23] Kroenke K, Spitzer RL, Williams JBW. The PHQ-9: validity of a brief depression severity measure. J Gen Intern Med. 2001;16:606–13. 10.1046/j.1525-1497.2001.016009606.x. 11556941 10.1046/j.1525-1497.2001.016009606.x PMC1495268

[24] Henrich G, Herschbach P. Fragen zur Lebenszufriedenheit (FLZ-M). In: Ravens-Sieberer U, Cieza A, Bullinger M, von Steinbuechel N, Poeppel E, editors. Lebensqualität und Gesundheitsökonomie in der Medizin. Konzepte, Methoden, Anwendung. Landsberg: ecomed; 2000. p. 98–110.

[25] Lukat J, Margraf J, Lutz R, van der Veld WM, Becker ES. Psychometric properties of the Positive Mental Health Scale (PMH-scale). BMC Psychol. 2016;4(1):8. 10.1186/s40359-016-0111-x. 26865173 10.1186/s40359-016-0111-x PMC4748628

[26] Fydrich T, Sommer G, Brähler E. Fragebogen zur sozialen Unterstützung (F-SozU). Göttingen: Hogrefe; 2007.

[27] Hahlweg K, Schulz W. Längsschnittliche psychometrische Analysen der Kurzform des „Fragebogens zur Beziehungszufriedenheit (FBZ-K)“: Befunde zur Stabilität und Konstruktvalidität. Diagnostica. 2023;69:156–68. 10.1026/0012-1924/a000312.

[28] Schumacher J, Leppert K, Gunzelmann T, Strauß B, Brähler E, et al. Die Resilienzskala – Ein Fragebogen zur Erfassung der psychischen Widerstandsfähigkeit als Personenmerkmal. Z Klin Psychol Psychiatr Psychother. 2005;2005(53):16–39.

[29] Propp O, Schulz W, Foran H. Sexuelles Risikoverhalten im Jugendalter. Familiäre und individuelle Faktoren. Kindh Entwickl. 2016;25:122–9. 10.1026/0942-5403/a000195.

[30] Rumpf HJ, Hapke U, John U. LAST. Lübecker Alkoholabhängigkeits- und -missbrauchs-Screening-Test (Testmanual). Göttingen: Hogrefe; 2001.

[31] Adamson SJ, Sellman JD. A prototype screening instrument for cannabis use disorder: the Cannabis Use Disorders Identification Test (CUDIT) in an alcohol-dependent clinical sample. Drug Alcohol Rev. 2003;22:309–15. 10.1080/0959523031000154454. 15385225 10.1080/0959523031000154454

[32] Esser G, Schmidt MH. Die Mannheimer Risikokinderstudie. Kindh Entwickl. 2017;26:198–202. 10.1026/0942-5403/a000232.

[33] Ravens-Sieberer U, Wille N, Bettge S, Erhart M. Psychische Gesundheit von Kindern und Jugendlichen in Deutschland. Ergebnisse aus der BELLA-Studie im Kinder- und Jugendgesundheitssurvey (KiGGS). Bundesgesundheitsbl Gesundheitsforsch Gesundheitsschutz. 2007;50:871–8. 10.1007/s00103-007-0250-6. 10.1007/s00103-007-0250-6 17514473

[34] Scheithauer H, Niebank K, Petermann F. Biopsychosoziale Risiken in der Entwicklung: Das Risiko- und Schutzfaktorenkonzept aus entwicklungspsychopathologischer Sicht. In: Petermann F, Niebank K, Scheithauer H, editors. Risiken in der frühkindlichen Entwicklung. Entwicklungspsychopathologie der ersten Lebensjahre (S. 65–97). Göttingen: Hogrefe; 2000. p. 65–97.

[35] Lovibond PF, Lovibond SH. Manual for the depression anxiety stress scales. Sydney: Psychology Foundation; 1995.

[36] Bäse B. Die sozial-räumliche Gliederung der Stadt Braunschweig. Methodik und Durchführung sozial-geographischer Analyse im städtischen Wohnumfeld auf der Grundlage des Zensus 1987. Braunschweig: Technische Universität Braunschweig; 1995.

[37] Arbeitsgruppe Deutsche Child Behavior Checklist. Elternfragebogen für Klein- und Vorschulkinder (CBCL 1 ½ -5). Köln: Arbeitsgruppe Kinder-, Jugend- und Familiendiagnostik (KJFD); 2000.

[38] Hayes AF. Introduction to mediation, moderation, and conditional process analysis: a regression-based approach. New York: Guilford Press; 2013.

[39] Loeb S, Bridges M, Bassok D, Fuller B, Rumberger RW. How much is too much? The influence of preschool centers on children’s social and cognitive development. Econ Educ Rev. 2007;26:52–66. 10.1016/j.econedurev.2005.11.005.

[40] Hölling H, Erhart M, Ravens-Sieberer U, Schlack R. Verhaltensauffälligkeiten bei Kindern und Jugendlichen. Erste Ergebnisse aus dem Kinder- und Jugendgesundheitssurvey (KiGGS). Bundesgesundheitsbl Gesundheitsforsch Gesundheitsschutz. 2007;50:784–93. 10.1007/s00103-007-0241-7. 10.1007/s00103-007-0241-7 17514464

[41] BMI/BAMF – Bundesministerium des Innern und für Heimat/Bundesamt für Migration und Flüchtlinge. Migrationsbericht der Bundesregierung. Migrationsbericht 2021. Berlin/Nürnberg: BMI/BAMF; 2023. https://www.bamf.de/SharedDocs/Anlagen/DE/Forschung/Migrationsberichte/migrationsbericht-2021.pdf?__blob=publicationFile&v=15. Accessed 04.01.2024.

[42] Statista Research Department. Alleinerziehende in Deutschland. 2024. https://de.statista.com/themen/5182/alleinerziehende-in-deutschland/#topicOverview.

[43] Hahlweg K, Ditzen B, Job A-K, Schulz W, Supke M, Walper S. COVID-19: Psychologische Folgen für Familie, Kinder und Partnerschaft. Z Klin Psychol Psychother. 2020;49:157–71. 10.1026/1616-3443/a000592.

