# Supplementary material for: Does early non-familial child care impact the development of mental health problems, risk behaviors, and educational outcomes until young adulthood?—Findings from an 18-year longitudinal study
Source: BMC Psychol. 2026 May 5;14:655. doi: 10.1186/s40359-026-04660-w (PMC13141441; doi:10.1186/s40359-026-04660-w)
Supplement: Supplementary file 1 — Supplementary Material 1. [file 40359_2026_4660_MOESM1_ESM.doc]

**Supplemental online material**

**Table A1 Dropout Analysis**

| Sample Characteristics | | Initial sample (N=280) | | | | | Participant FU18 (N=225) | | | | | Dropouts  (N=55) | | | | | χ^2^-Score | | p |
| --- | --- | --- | --- | --- | --- | --- | --- | --- | --- | --- | --- | --- | --- | --- | --- | --- | --- | --- | --- |
|  |  | N | | % | | | N | | % | | | N | | % | | |  |  |  |
| Family status (Pre):  - single parent  - two-parent family | | 61  219 | | 21.8  78.2 | | | 41  184 | | 18.2  81.8 | | | 20  35 | | 36.4  63.6 | | | 8.536 | | .003** |
| Migration background (Pre):  - Migration background  - No migration background | | 27  253 | | 9.6  90.4 | | | 24  201 | | 10.7  89.3 | | | 3  52 | | 5.5  94.5 | | | 1.378 | | .240 |
| Educational level of mothers (Pre):  - Without school leaving certificate. 9 classes  - 10 classes  - A-Levels/High school | | 31  96  152 | | 11.1  34.4  54.5 | | | 16  78  130 | | 7.1  34.8  58.0 | | | 15  18  22 | | 27.3  32.7  40.0 | | | 18.798 | | <.001*** |
| Highest school degree of fathers (Pre):  - Without school leaving certificate. 9 classes  - 10 classes  - A-Levels/High school | | 33  50  136 | | 15.2  22.8  62.1 | | | 21  47  116 | | 11.4  25.6  63.0 | | | 12  3  20 | | 34.3  8.6  57.1 | | | 14.046 | | <.001*** |
| Sex (Pre)  - female  - male | | 136  144 | | 48.6  51.4 | | | 111  114 | | 49.3  50.7 | | | 26  29 | | 47.3  52.7 | | | 0.075 | | .784 |
| Monthly household income (German Mark; DM) (Pre)  - < 3000  - 3000-6000  - > 6000 | | 52  138  83 | | 19.0  50.5  30.4 | | | 35  111  73 | | 16.0  50.7  33.3 | | | 17  27  10 | | 31.5  50.0  18.5 | | | 8.595 | | .014* |
|  | initial sample (N=280) | | | | | Participant FU18 (N=225) | | | | | Dropouts  (N=55) | | | | | T-Score | | p | |
|  | N | | M | | SD | N | | M | | SD | N | | M | | SD |  |  |  |  |
| Age of child (Pre) | 280 | | 4.1 | | 1.0 | 225 | | 4.0 | | 1.0 | 55 | | 4.2 | | 1.1 | .952 | | .342 | |
| Age of mothers (Pre) | 279 | | 35.2 | | 5.0 | 224 | | 35.6 | | 4.7 | 55 | | 33.9 | | 5.9 | -2.193 | | .029* | |
| Age of fathers (Pre) | 220 | | 38.8 | | 6.0 | 185 | | 38.5 | | 5.5 | 35 | | 40.0 | | 8.4 | 1.288 | | .199 | |
| Number of siblings (Pre) | 280 | | 1.1 | | 0.9 | 225 | | 1.1 | | 0.9 | 55 | | 1.1 | | 1.0 | .278 | | .963 | |

*Notes.* ^+^*p*<.10. **p*<.05. ***p*<.01. ****p*<.001

**Table A2 Risk Index: Statistical Parameters und correlations with Child Mental Health Problems. Protective Factors. Risk Behaviors. Education Level and Vocational**

| Statistical Parameters |  | |
| --- | --- | --- |
| M  SD  range | 1.82  1.33  0 – 7 |  |
| distribution  0  1  2  ≥3 | N  22  88  49  55 | %  10.3  41.1  22.9  27.7 |
|  | Risk Index | |
| Correlations | r | p |
| ASR Internalizing | .041 | .568 |
| ASR Externalizing | .085 | .231 |
| Anxiety symptoms (GAD-7) | .128 | .071^+^ |
| Depressive Symptoms (PHQ-9) | .036 | .615 |
| Life satisfaction (LSS) | .013 | .858 |
| Positive mental health (PHM) | -.149 | .037* |
| Social support (SSQ) | -.137 | .054^+^ |
| Relationship quality (ADAS) | .049 | .646 |
| Resilience (RS-11) | -.084 | .236 |
| Risky sexual behavior | .062 | .390 |
| Alcohol abuse (LAST) | .093 | .193 |
| Tobacco use | .011 | .881 |
| Cannabis use (CUDIT) | .126 | .079^+^ |
| Alcohol abuse (LAST) | -.252 | <.001*** |
| Vocational attainment | -.229 | <.001*** |

*Notes.* ^+^*p*<.10. **p*<.05. ***p*<.01. ****p*<.001

**Table A3 Differences Between Parental and Non-Familial Early Childhood Care (NFECC): Child Mental Health Problems. Protective Factors. Risk Behaviors. Education Level and Vocational Attainment in Young Adulthood (FU18. Analysis of Covariance with Risk Index and Participation in Triple P at Pre as a Covariate and Effect Sizes) – Benjamini-Hochberg correction**

| **Variables** | **p** | **Rank** | **(i/m x Q)** | **significance** |
| --- | --- | --- | --- | --- |
| Cannabis use (CUDIT) | .002 | 1 | .003 | sign |
| Tobacco use | .005 | 2 | .007 | sign |
| Alcohol abuse (LAST) | .011 | 3 | .010 | sign |
| Risky sexual behavior | .013 | 4 | .013 | sign |
| Vocational attainment | .016 | 5 | .017 | sign |
| Relationship quality (ADAS) | .039 | 6 | .020 | - |
| ASR Externalizing | .158 | 7 | .023 | - |
| Education level | .239 | 8 | .027 | - |
| Depressive Symptoms (PHQ-9) | .419 | 9 | .030 | - |
| Positive mental health (PHM) | .476 | 10 | .033 | - |
| Social support (SSQ) | .633 | 11 | .037 | - |
| Anxiety symptoms (GAD-7) | .689 | 12 | .040 | - |
| ASR Internalizing | .860 | 13 | .043 | - |
| Resilience (RS-11) | .893 | 14 | .047 | - |
| Life satisfaction (LSS) | .996 | 15 | .050 | - |

*Notes.* I=Rank of p-value; m=5 (number of tests Tests); Q=0.05 (False Discovery Rate; sign= significant

**Table A4 Results of the Slope Analyses of Significant Interaction Effects in Moderator Analyses with the Predictor Non-Familial Early Childcare and the Moderator Migration Background (MB)**

| Criterion | Moderator | Familial Care | | Non-Familial Care | |  |  |  |
| --- | --- | --- | --- | --- | --- | --- | --- | --- |
|  |  | *M* | *SD* | *M* | *SD* | *B* | *t* | *p* |
| Depressive symptoms (PHQ-9) | No MB | 5.87 | 4.74 | 5.81 | 5.11 | -0.06 | -0.08 | .940 |
|  | With MB | 3.75 | 2.66 | 10.00 | 5.01 | 6.34 | 2.81 | .006** |
| Life satisfaction (LSS) | No MB | 53.15 | 27.28 | 58.45 | 34.70 | 5.31 | 1.01 | .313 |
|  | With MB | 68.63 | 49.46 | 33.09 | 33.28 | -35.53 | -2.36 | .019* |
| Positive mental health (PMH) | No MB | 28.25 | 6.14 | 29.38 | 5.92 | 1.13 | 1.16 | .246 |
|  | With MB | 29.38 | 2.20 | 24.27 | 5.90 | -5.10 | -1.86 | .065^+^ |
| Resilience (RS-11) | No MB | 56.92 | 8.75 | 57.78 | 10.85 | 0.86 | -0.52 | .603 |
|  | With MB | 60.38 | 6.44 | 50.91 | 15.32 | -9.47 | -2.00 | .047* |
| Sexual risk behavior | No MB | 1.89 | 2.24 | 2.60 | 2.22 | 0.71 | 1.96 | .052^+^ |
|  | With MB | 0.63 | 0.52 | 3.18 | 2.86 | 2.56 | 2.47 | .015* |
| Tobacco use | No MB | 0.33 | 0.48 | 0.49 | 0.50 | 0.64 | 1.92 | .054^+^ |
|  | With MB | 0.38 | 0.52 | 1.00 | 0.00 | 15.71 | 0.03 | .979 |

*Notes.* ^+^*p*<.10. **p*<.05. ***p*<01. ****p*<.001.

**Table A5 Results of Moderator Analyses at FU18. Migration background – Benjamini-Hochberg correction**

| **Variables** | **p** | **Rank** | **(i/m x Q)** | **significance** |
| --- | --- | --- | --- | --- |
| Depressive Symptoms (PHQ-9) | .008 | 1 | .003 | - |
| Life satisfaction (LSS) | .011 | 2 | .007 | - |
| Positive mental health (PHM) | .034 | 3 | .010 | - |
| Resilience (RS-11) | .041 | 4 | .013 | - |
| Tobacco use | .046 | 5 | .017 | - |
| ASR Internalizing | .082 | 6 | .020 | - |
| Risky sexual behavior | .095 | 7 | .023 | - |
| Anxiety symptoms (GAD-7) | .102 | 8 | .027 | - |
| Education level | .135 | 9 | .030 | - |
| ASR Externalizing | .152 | 10 | .033 | - |
| Cannabis use (CUDIT) | .224 | 11 | .037 | - |
| Social support (SSQ) | .334 | 12 | .040 | - |
| Alcohol abuse (LAST) | .353 | 13 | .043 | - |
| Relationship quality (ADAS) | .459 | 14 | .047 | - |
| Vocational attainment | .550 | 15 | .050 | - |

*Notes.* I=Rank of p-value; m=5 (number of tests Tests); Q=0.05 (False Discovery Rate); sign= significant

**Table A6** Results of Slope Analyses for Significant Interaction Effects in Moderator Analyses with the Predictor Non-Familial Early Childcare and the Moderator Socioeconomic Status (SES)

| Criterion | Moderator^1^ | Familial Care | | NFECC | |  |  |  |
| --- | --- | --- | --- | --- | --- | --- | --- | --- |
|  |  | *M* | *SD* | *M* | *SD* | *B* | *t* | *p* |
| Life satisfaction (LSS) | Middle SES | 57.68 | 35.39 | 47.74 | 35.94 | -9.94 | -0.97 | .340 |
|  | High SES | 53.98 | 25.16 | 55.10 | 32.17 | 1.12 | 0.21 | .835 |
| Alcohol abuse (LAST) | Middle SES | 0.46 | 0.66 | 1.59 | 1.53 | 1.13 | 3.31 | .002** |
|  | High SES | 0.74 | 0.98 | 1.15 | 1.32 | 0.40 | 1.85 | .066^+^ |

*Notes.*^1^ Due to the limited sample size. a slope analysis for the lower socioeconomic status was not conducted. ^+^*p*<.10. **p*<.05. ***p*<.01. ****p*<.001.

**Table A7 Results of Moderator Analyses at FU18. Socioeconomic status – Benjamini-Hochberg correction**

| **Variables** | **p** | **Rank** | **(i/m x Q)** | **significance** |
| --- | --- | --- | --- | --- |
| Alcohol abuse (LAST) | .001 | 1 | .003 | sign |
| Life satisfaction (LSS) | .026 | 2 | .007 | - |
| Positive mental health (PHM) | .123 | 3 | .010 | - |
| Education level | .170 | 4 | .013 | - |
| Anxiety symptoms (GAD-7) | .187 | 5 | .017 | - |
| Vocational attainment | .191 | 6 | .020 | - |
| Depressive Symptoms (PHQ-9) | .240 | 7 | .023 | - |
| ASR Internalizing | .261 | 8 | .027 | - |
| Tobacco use | .321 | 9 | .030 | - |
| Resilience (RS-11) | .344 | 10 | .033 | - |
| Social support (SSQ) | .445 | 11 | .037 | - |
| ASR Externalizing | .480 | 12 | .040 | - |
| Relationship quality (ADAS) | .904 | 13 | .043 | - |
| Cannabis use (CUDIT) | .904 | 14 | .047 | - |
| Risky sexual behavior | .909 | 15 | .050 | - |

*Notes.* I=Rank of p-value; m=5 (number of tests Tests); Q=0.05 (False Discovery Rate); sign= significant

**Table A8 Results of the Univariate Linear Regression Models in the Subgroup of Non-Familial Early Childhood Care (NFECC) Children with Age of Entry into Non-Familial Care as a Predictor and the Individual Risk Index and Participation in Triple P at Pre as a Covariate and Effect Sizes – Benjamini-Hochberg correction**

| **Variables** | **p** | **Rank** | **(i/m x Q)** | **significance** |
| --- | --- | --- | --- | --- |
| Relationship quality (ADAS) | .019 | 1 | .003 | - |
| Social support (SSQ) | .030 | 2 | .007 | - |
| ASR Internalizing | .051 | 3 | .010 | - |
| Resilience (RS-11) | .052 | 4 | .013 | - |
| Depressive Symptoms (PHQ-9) | .054 | 5 | .017 | - |
| Positive mental health (PHM) | .057 | 6 | .020 | - |
| Education level | .063 | 7 | .023 | - |
| Anxiety symptoms (GAD-7) | .076 | 8 | .027 | - |
| ASR Externalizing | .147 | 9 | .030 | - |
| Life satisfaction (LSS) | .331 | 10 | .033 | - |
| Risky sexual behavior | .340 | 11 | .037 | - |
| Tobacco use | .358 | 12 | .040 | - |
| Cannabis use (CUDIT) | .713 | 13 | .043 | - |
| Alcohol abuse (LAST) | .845 | 14 | .047 | - |
| Vocational attainment | .894 | 15 | .050 | - |

*Notes.* I=Rank of p-value; m=5 (number of tests Tests); Q=0.05 (False Discovery Rate); sign= significant
